# Chilling and Heat Stress-Induced Physiological Changes and MicroRNA-Related Mechanism in Sweetpotato (*Ipomoea batatas* L.)

**DOI:** 10.3389/fpls.2020.00687

**Published:** 2020-05-26

**Authors:** Jingjing Yu, Dan Su, Dongjing Yang, Tingting Dong, Zhonghou Tang, Hongmin Li, Yonghua Han, Zongyun Li, Baohong Zhang

**Affiliations:** ^1^Institute of Integrative Plant Biology, School of Life Sciences, Jiangsu Normal University, Xuzhou, China; ^2^Jiangsu Key Laboratory of Phylogenomics and Comparative Genomics, Jiangsu Normal University, Xuzhou, China; ^3^Department of Biology, East Carolina University, Greenville, NC, United States; ^4^Xuzhou Institute of Agricultural Sciences in Xuhuai District, Jiangsu Xuzhou Sweetpotato Research Center, Sweet Potato Research Institute, CAAS, Xuzhou, China; ^5^Key Laboratory of Biology and Genetic Improvement of Sweetpotato, Ministry of Agriculture, Xuzhou, China

**Keywords:** sweetpotato, chilling, heat stress, miRNA, oxidative stress

## Abstract

Sweetpotato (*Ipomoea batatas* (L.) Lam.) is an important industrial and food crop. Both chilling and heat stress inhibits sweetpotato growth and development and then affects yield. However, the physiological and molecular mechanisms of sweetpotato response to chilling and heat stress is unclear. In this study, we investigated the effect of extreme temperature on sweetpotato physiological response, with a focus on oxidative stress and the potential microRNA (miRNA)-mediated molecular mechanism. Our results showed that both chilling and heat stress resulted in accumulation of reactive oxygen species (ROS), including H_2_O_2_ and O_2_^–^, and caused oxidative stress in sweetpotato. This further affected the activities of oxidative stress-related enzymes and products, including SOD, POD, and MDA. Both chilling and heat stress inhibited POD activities but induced the enzyme activities of SOD and MDA. This suggests that sweetpotato cells initiated its own defense mechanism to handle extreme temperature-caused oxidative damage. Oxidative damage and repair are one mechanism that sweetpotato plants respond to extreme temperatures. Another potential mechanism is miRNA-mediated gene response. Chilling and heat stress altered the expression of stress-responsive miRNAs in sweetpotato seedlings. These miRNAs regulate sweetpotato response to extreme stress through targeting individual protein-coding genes.

## Introduction

Both chilling and heat stresses dysregulate active oxygen metabolism in plants, which leads to many cellular and physical changes, including oxidative stress, cell membrane lipid peroxidation, protein denaturation and nucleotide damage; the damage caused by the stress may also cause cell death (Kuk et al., 2003). To avoid the damage caused by oxidative stress, during the long history of evolution, plants develop their own antioxidant system to regulate the levels of reactive oxygen species (ROS). With increase of heat stress, the plasma membrane permeability was increased in the leaves of four Lysimachia plants, and the activities of SOD and POD were first increased and then decreased, while the contents of chlorophyll, soluble protein, and proline were decreased (Xu and Zhang, 2009). The study performed by Ren et al. (2012) showed that after treatment with heat stress for 24 h, the SOD activity was significantly higher in treatment groups than that in the leaves of controls; however, POD activities were lower than that in the controls. Li et al. (2015) observed that SOD, POD, catalase (CAT) activity and malondialdehyde (MDA) content were increased in the leaves of *Atractylodes lancea* with the prolongation of heat stress. Under chilling stress, the activities of CAT, POD and SOD and MDA content were increased in potato leaves; at the same time, chilling stress also increased the contents of soluble sugar, protein, and proline (Xu et al., 2016). However, as increasing the chilling treated time, the changes in physiological indicators showed different trends. Wang et al. (2010) observed that chilling treatment affected membrane lipid peroxidation and antioxidant enzyme activities in *Capsicum annuum* seedlings. There are also several studies on the impact of temperature stress on sweetpotato gene expression. These studies show that low temperature treatment induced aberrant expression of many coded and non-coded genes (Ji et al., 2017a, 2019, 2020; Xie et al., 2017, 2019). Several studies over-expressed an individual gene to enhance the tolerance to low temperature stress in sweetpotato (Kim et al., 2011; Fan et al., 2012; Ji et al., 2017b; Jin et al., 2017). Wang et al. (2019) also recently studied the antioxidative system in sweetpotato root under low temperature storage condition; their result showed that the activities of antioxidant enzymes were changed quickly during sweetpotato storage under chilling stress (Wang et al., 2019).

MicroRNAs (miRNAs) are an abundant class of endogenous non-coding small regulated RNAs that play a critical role in gene regulatory networks at the post-transcription levels (Zhang B. et al., 2007). miRNAs are widely existed and highly conserved in plants. miRNAs regulate plant growth and stress tolerance by complementing the mRNA sequence of a target gene, mediating the RNA-indcued silencing complex (RISC) to degrade the mRNA of a target gene or inhibit its translation (Li and Zhang, 2016). In recent years, with the rapid development of sequencing technology, molecular biology and bioinformatics, research on small non-coding RNA has become a research hotspot (Zhang and Unver, 2018). miRNAs not only participate in the regulation of plant growth and development, but also in plant response to various abiotic stresses, including chilling, salinity, heat, drought, and oxidative stresses (Zhang, 2015). There are more and more reports on the regulation of conserved miRNAs for plant stress adaptation. However, miRNAs are differentially expressed in different plant species, tissues, and stress. Under chilling stress, three conserved miRNAs with significant expression changes and 25 new candidate miRNAs were involved in *Brachypodium* response to low temperature treatment (Zhang et al., 2009). When wheat and barley were subjected to heat stress, several miRNAs, including miRl60, miRl66, and miRl67, show aberrant expression (Xin et al., 2010). In *Arabidopsis thaliana*, miR156 responded to heat stress by targeting on *SPL* transcription factor (Stief et al., 2014). The expression of miR397a and miR171 was induced by heat stress in the leaves and roots of *A. thaliana* (Mahale et al., 2014), and it has been demonstrated that miR397 participated in heat stress by regulating the expression of L-ascorbate oxidase (Jeong et al., 2012). Xie et al. (2017) identified 190 conserved miRNAs and 191 novel miRNAs associated with chilling stress in sweetpotato. Although certain stress-responsive miRNAs are highly conserved among different plant species, there are also some species-specific miRNAs such as miR403 that respond to chilling stress only in a specific plant species (Zeng et al., 2010).

Sweetpotato (*Ipomoea batatas* (L.) Lam.) is an important industrial and food crop, and it is widely planted around the world. However, sweetpotato is very sensitive to temperature change. Both heat and chilling temperature potentially affects sweetpotato growth and development and further affect sweetpotato yield and biomass. Although there are several reports on impact of extreme temperatures on sweetpotato (Kim et al., 2011; Fan et al., 2012; Ji et al., 2017a, 2019, 2020; Xie et al., 2017, 2019; Wang et al., 2019), but these studies majorly focused on gene expression analysis and the impact during the sweetpotato storage. There is few study on the impact of temperature stress on sweetpotato seedlings, particularly on the oxidative stress. Additionally, the physiological and molecular mechanisms for the chilling and heat-induced damages are unclear in sweetpotato. In this study, we investigated the physiological changes, particularly oxidative stress, in sweetpotato seedlings during chilling and heat stress; then, we studied the expression profiles of selected miRNAs and their targets to elucidate the potential miRNA-mediated mechanism during sweetpotato response to these two aberrant temperature treatments. Our results provide an important scientific foundation for breeding high tolerant sweetpotato to heat and chilling stress as well as better storage of sweetpotato and better agricultural practices for sweetpotato cultivation.

## Materials and Methods

### Sweetpotato Culture and Temperature Treatments

Sweetpotato [*Ipomoea batatas* (L.) Lam.] cv. Covington was grown and maintained in the greenhouse, which is bred by North Carolina State University and are currently widely cultivated in the United States. The seedlings at same age and size (about 6 cm in height) were selected, cut and re-planted in the 7 × 7 × 7 pots with commercially artificial soil. Traditional practices, including watering, was performed daily. After 2 weeks of culture in the greenhouse, all the seedlings generated roots and grew well. The seedlings with similar growth were transferred to the growth incubators for temperature treatments. The seedling plants were divided into three groups and they were cultured at 4, 25, and 47°C, respectively. 4 and 47°C represented chilling and heat treatment and 25°C served as controls. 4°C were the temperature commonly used for the low temperature treatment (Ji et al., 2020; Vyse et al., 2020). Sweetpotato grows at summer hot season, it may suffer from the 40s°C. To study the impact of extreme hot temperature, we treated sweetpotato seedlings using 47°C. Each group had a total of 60 plants. To study the potential impact of extreme temperature, after 6, 12, 24, and 48 h of treatment, the first fully expanded leaves were collected from each treatment. For physiological and biochemical analysis, five biological replicates were collected for each treatment at each time point. For gene expression analysis, at least three biological replicates were collected, and the samples were immediately frozen at liquid nitrogen and then stored at −80°C.

### Physiological and Biochemical Analysis

#### Analysis of H_2_O_2_ Accumulation

The analysis of H_2_O_2_ accumulation was carried out using a 3,3′-diaminobenzidine (DAB) staining method according to a previous report (Thordal-Christensen et al., 1997). Briefly, plant leaves were individually immersed in 1 mg/ml DAB solution (PH 3.8), and then incubated in an incubator at 28°C for 8 h. After removing the staining solution, 95% ethanol was added for 24 h to remove chlorophyll. The brown spots on the leaves present the accumulation of H_2_O_2_. The more brown color represented the more H_2_O_2_ accumulation. Five biological replicates were run for each treatment and control.

#### Enzyme Extraction and Analysis

One gram of fresh leaves was homogenized on ice in 5 mL of 50 mM sodium phosphate buffer (pH 7.8). The homogenate was centrifuged at 10,000 rpm for 20 min at 4°C. The supernatants were used for analyzing superoxide dismutase (SOD) and peroxidase (POD) enzyme activity and the atomic oxide radical anion (O_2_^–^•) content. Five biological replicates were run for each treatment and control.

Superoxide dismutase activity assay was performed according to a previous report (He et al., 2009). Briefly, the crude extract was added to the reaction mixture including 50 mM sodium phosphate buffer (pH 7.8), 0.75 mM nitroblue tetrazolium (NBT), 26 mM methionine, 0.02 mM riboflavin, and 1 μM ethylenediaminetetraacetic acid (EDTA). The enzyme unit is to inhibit 50% of NBT photochemical reduction.

Peroxidase activity assay was performed according to a previous report (Hammerschmidt et al., 1982). Briefly, the crude extract was used to react with a solution mixture, including 0.2 M sodium phosphate buffer (pH 6.0), 0.3% guaiacol and hydrogen peroxide (H_2_O_2_). The oxidation of guaiacol was monitored by the increase in absorbance at 470 nm for 1 min with a spectrophotometer.

The atomic oxide radical anion (O_2_^–^•) content assay was measured according the method of a previous report (Wang and Luo, 1990). The supernatant was added to a solution containing 50 mM sodium phosphate buffer and 10 mM hydroxylamine hydrochloride. The reaction was performed at 25°C for 30 min. Then, 17 mM sulfanilic acid 1 mL and 17 mM 1-naphthylamine solution 1 mL were added into the reaction and mixed thoroughly; continuously kept the reaction at 25°C for another 20 min. The absorbance was measured at 530 nm with a UV spectrophotometer.

Malondialdehyde (MDA) analysis was performed according to previous studies (Heath and Packer, 1968; López-Serrano et al., 2019). Briefly, one gram of fresh leaves was ground in 10 mL of 5% trichloroacetic acid buffer mixed with quartz sand. Homogenates were centrifuged at 3,000 rpm for 15 min. 0.5% TBA was added to the extract, and the mixture was heated at 100°C for 15 min and then quickly transferred to an ice bath to block the reaction. The cooled mixture was centrifuged at 3,000 *g* for 10 min. The absorbance of the supernatant was recorded at 532 nm and 600 nm with a UV spectrophotometer.

#### RNA Isolation and Gene Expression Analysis

Total RNAs were isolated from the leaves of each treatment and control at each time point using the mirPremier^®^ microRNA Isolation Kit (Sigma) following the supplier’s instructions. The purified 1000 ng RNA was reverse-transcribed to cDNA using the TaqMan^®^ MicroRNA Reverse Transcription Kit (Applied Biosystems). Quantitative real-time PCR was employed to analyze the expression of 26 miRNA and their 14 targets by using SensiFAST^®^ SYBR HI-ROX Kit (Bioline) on a 7300 Real Time PCR System (Applied Biosystems).

To explore the regulatory role of miRNAs and their target genes in sweetpotato response to chilling and high temperature stress, we analyzed the expression profiles of 26 miRNAs and 14 target genes. The 26 miRNAs were IbmiR156, IbmiR160, IbmiR164, IbmiR166, IbmiR172, IbmiR390, IbmiR395, IbmiR397, IbmiR857, IbmiR171, IbmiR159, IbmiR162, IbmiR165, IbmiR167, IbmiR169, IbmiR2119, IbmiR319, IbmiR398, IbmiR403, IbmiR408, IbmiR827, IbmiR847, IbmiR858, IbmiR396, IbmiR862, and IbmiR393. These miRNAs were selected based on our previous studies (Xie et al., 2017) and other studies in other plant species (Zhang, 2015), these miRNAs were classified to: (1) miRNAs are associated with plant response to different environmental stresses, including chilling and high temperature stress, and (2) miRNAs are associated with plant growth and development. However, no study has shown how these miRNAs response to chilling and heat stresses in sweetpotato seedlings. A total of 14 miRNA target genes were also analyzed and their corresponding miRNA were *IbMAA (IbmiR403), IbAP2 (IbmiR172)*, *IbARF10 (IbmiR160)*, *IbARF8 (IbmiR167)*, *IbATHB (IbmiR166)*, *IbCNR8 (IbmiR156)*, *IbDCL1 (IbmiR162)*, *IbMYB (IbmiR159)*, *IbKPNB (IbmiR166)*, *IbNFYA (IbmiR169)*, *IbSPL15 (IbmiR156)*, *IbSPL2 (IbmiR156)*, *IbTCP2 (IbmiR159, IbmiR319)*, *IbZAT (IbmiR403)*, respectively. Both gene information and the primer sequences were list in Table 1 for miRNAs and Table 2 for protein-coding gene, respectively. The following temperature program was used: 95°C for 10 min, followed by the 40 amplification cycles at 95°C for 15 s and 60°C for 60 s. Sweetpotato *elf* gene was used as a reference gene. Each treatment or control had three biological replicates with three technological replicates.

**TABLE 1:** Twenty six selected miRNAs and the primers *.

miRNA	miRNA sequence	RT primer	Forward primer
IbmiR156	TGACAGAAGAGAGTGAGCAC	GTCGTATCCAGTGCAGGGTCCGAGG	GCGGCGGTGACAGAAGAGAGTG
		TATTCGCACTGGATACGACGTGCTC	
IbmiR159	TTTGGATTGAAGGGAGCTCTA	GTCGTATCCAGTGCAGGGTCCGAGG	GCGGCGGTTTGGATTGAAGGGAG
		TATTCGCACTGGATACGACTAGAGC	
IbmiR160	TGCCTGGCTCCCTGTATGCCA	GTCGTATCCAGTGCAGGGTCCGAGG	GCGGCGGTGCCTGGCTCCCTG
		TATTCGCACTGGATACGACTGGCAT	
IbmiR162	TCGATAAACCTCTGCATCCAG	GTCGTATCCAGTGCAGGGTCCGAGG	GCGGCGGTCGATAAACCTCTGC
		TATTCGCACTGGATACGACCTGGAT	
IbmiR164	TGGAGAAGCAGGGCACGTGCA	GTCGTATCCAGTGCAGGGTCCGAGG	GCGGCGGTGGAGAAGCAGGGCAC
		TATTCGCACTGGATACGACTGCACG	
IbmiR165	GGAATGTTGTCTGGATCGAGG	GTCGTATCCAGTGCAGGGTCCGAGG	GCGGCGGTCGGACCAGGCTTCATC
		TATTCGCACTGGATACGACCCTCGA	
IbmiR166	GGACTGTTGTCTGGCTCGAGG	GTCGTATCCAGTGCAGGGTCCGAGG	GCGGCGGTCGGACCAGGCTTC
		TATTCGCACTGGATACGACGGGGAA	
IbmiR167	TGAAGCTGCCAGCATGATCTA	GTCGTATCCAGTGCAGGGTCCGAGG	GCGGCGGTGAAGCTGCCAGCATG
		TATTCGCACTGGATACGACTAGATC	
IbmiR169	CAGCCAAGGATGACTTGCCGA	GTCGTATCCAGTGCAGGGTCCGAGG	GCGGCGGCAGCCAAGGATGACTTG
		TATTCGCACTGGATACGACTCGGCA	
IbmiR172	AGAATCTTGATGATGCTGCAT	GTCGTATCCAGTGCAGGGTCCGAGG	GCGGCGGAGAATCTTGATGATG
		TATTCGCACTGGATACGACATGCAG	
IbmiR2119	TCAAAGGGAGTTGTAGGGGAA	GTCGTATCCAGTGCAGGGTCCGAGG	GCGGCGGTCAAAGGGAGTTGTAG
		TATTCGCACTGGATACGACTTCCCC	
IbmiR319	TTGGACTGAAGGGAGCTCCCT	GTCGTATCCAGTGCAGGGTCCGAGG	GCGGCGGTTGGACTGAAGGGAG
		TATTCGCACTGGATACGACAGGGAG	
IbmiR390	AAGCTCAGGAGGGATAGCGCC	GTCGTATCCAGTGCAGGGTCCGAGG	GCGGCGGAAGCTCAGGAGGGATAG
		TATTCGCACTGGATACGACGGCGCT	
IbmiR395	CTGAAGTGTTTGGGGGAACTC	GTCGTATCCAGTGCAGGGTCCGAGG	GCGGCGGCTGAAGTGTTTGGGG
		TATTCGCACTGGATACGACGAGTTC	
IbmiR397	TCATTGAGTGCAGCGTTGATG	GTCGTATCCAGTGCAGGGTCCGAGG	GCGGCGGTCATTGAGTGCAGCG
		TATTCGCACTGGATACGACCATCAA	
IbmiR398	TGTGTTCTCAGGTCACCCCTT	GTCGTATCCAGTGCAGGGTCCGAGG	GCGGCGGTGTGTTCTCAGGTCAC
		TATTCGCACTGGATACGACAAGGGG	
IbmiR403	TTAGATTCACGCACAAACTCG	GTCGTATCCAGTGCAGGGTCCGAGG	GCGGCGGTTAGATTCACGCAC
		TATTCGCACTGGATACGACCGAGTT	
IbmiR408	ATGCACTGCCTCTTCCCTGGC	GTCGTATCCAGTGCAGGGTCCGAGG	GCGGCGGATGCACTGCCTCTTC
		TATTCGCACTGGATACGACGCCAGG	
IbmiR827	TTAGATGACCATCAACAAACT	GTCGTATCCAGTGCAGGGTCCGAGG	GCGGCGGTTAGATGACCATCAAC
		TATTCGCACTGGATACGACAGTTTG	
IbmiR847	TCACTCCTCTTCTTCTTGATG	GTCGTATCCAGTGCAGGGTCCGAGG	GCGGCGGTCACTCCTCTTCTTC
		TATTCGCACTGGATACGACCATCAA	
IbmiR857	TTTTGTATGTTGAAGGTGTAT	GTCGTATCCAGTGCAGGGTCCGAGG	GCGGCGGTTTTGTATGTTGAAG
		TATTCGCACTGGATACGACATACAC	
IbmiR858	TTTCGTTGTCTGTTCGACCTT	GTCGTATCCAGTGCAGGGTCCGAGG	GCGGCGGTTTCGTTGTCTGTTC
		TATTCGCACTGGATACGACAAGGTC	
IbmiR171	TGATTGAGCCGCGCCAATATC	GTCGTATCCAGTGCAGGGTCCGAGG	GCGGCGGTGATTGAGCCGCGCC
		TATTCGCACTGGATACGACGATATT	
IbmiR396	TTCCACAGCTTTCTTGAACTG	GTCGTATCCAGTGCAGGGTCCGAGG	GCGGCGGTTCCACAGCTTTCTTG
		TATTCGCACTGGATACGACCAGTTC	
IbmiR862	TCCAATAGGTCGAGCATGTGC	GTCGTATCCAGTGCAGGGTCCGAGG	GCGGCGGTCCAATAGGTCGAGC
		TATTCGCACTGGATACGACGCACAT	
IbmiR393	TCCAAAGGGATCGCATTGATCC	GTCGTATCCAGTGCAGGGTCCGAGG	GCGGCGGTCCAAAGGGATCGC
		TATTCGCACTGGATACGACGGATCA	

*The reverse primer is same: ATCCAGTGCAGGGTCCGAGG.*

**TABLE 2:** Fourteen miRNA target gene, one reference gene and their primers.

Gene name	Gene ID	Forward primer	Reversed primer
Ibelf*	XM_019343175.1	CCATCTCTTTGACGGCTGGTTG	TCTCTGCACGCTCAAGAAGG
IbAP2	>comp100367_c2	TGGGATGAAGGGTGCTGTTC	ATTCGACACCGATCCAACCC
IbARF10	itf10g17680	GTCACGACCAGCGTTCTTCA	GGCTGAAAGGGATTGCTTCG
IbARF8	itf08g06430	AGTCGGCTCCTAAGTCCTCC	TCGAACCGCTAGGTTTGTCC
IbATHB	>comp1489_c1	AGCTGGCCTTCTCGCAATAG	AATCCGGACCAGGCTTCATC
IbCNR8	>comp78842_c1	ACGAAACGAGAACCAGGGAG	TGTGTATTGGGAGGTGTGGC
IbDCL1	>comp62741_c1	GACATTCTCCAGGGTGGGTG	TCATTGCCAAACAGCACAGC
IbMYB	itf15g01410	TGCGTAATAGCCAGATGGGC	TCCTCCTTGAAGTCCAGTGC
IbKPNB	>comp25211_c2	GTGGCCATTGCCTCAAACTG	CACTGGGCAGTAATGCTGGT
IbMAA	>comp102512_c2	TTTCAGCGAGCAAATGTGGC	ATCAAAGTCGCACCATTGCC
IbNFYA	>comp27679_c3	AGCTATGGAAGCCGATGCTG	GCACCCGAGATCCATACACG
IbSPL15	>comp20044_c1	ATGGATTTCGCCTCGTACCC	TAGCAGCATCCGAACCTAGC
IbSPL2	>comp104349_c6	TGGGATGAAGGGTGCTGTTC	ATTCGACACCGATCCAACCC
IbTCP2	itf02g19880	CCTAGTCAGCAACTCGGCTC	CCCGCAAACATGCCTAACTG
IbZAT	>comp93639_c2	TTCTTCACCACAGGAACGCC	TGGAGTTCGCCATTGGACAG

* reference gene.

#### Statistical Analysis

Each treatment and control had at least three biological replicates for each measured trait at each time point. ANOVA was performed to analyze the significance between different treatment and controls at each time point. If *p*-value was less than 0.01, it was considered extremely significant difference and used ^∗∗^ to show the extreme significance. If *p*-value was less than 0.05, it was considered significant difference and used ^∗^ to show the significance.

## Results

### Effect of Chilling and Heat Treatment on Sweetpotato Growth

**FIGURE 1:**
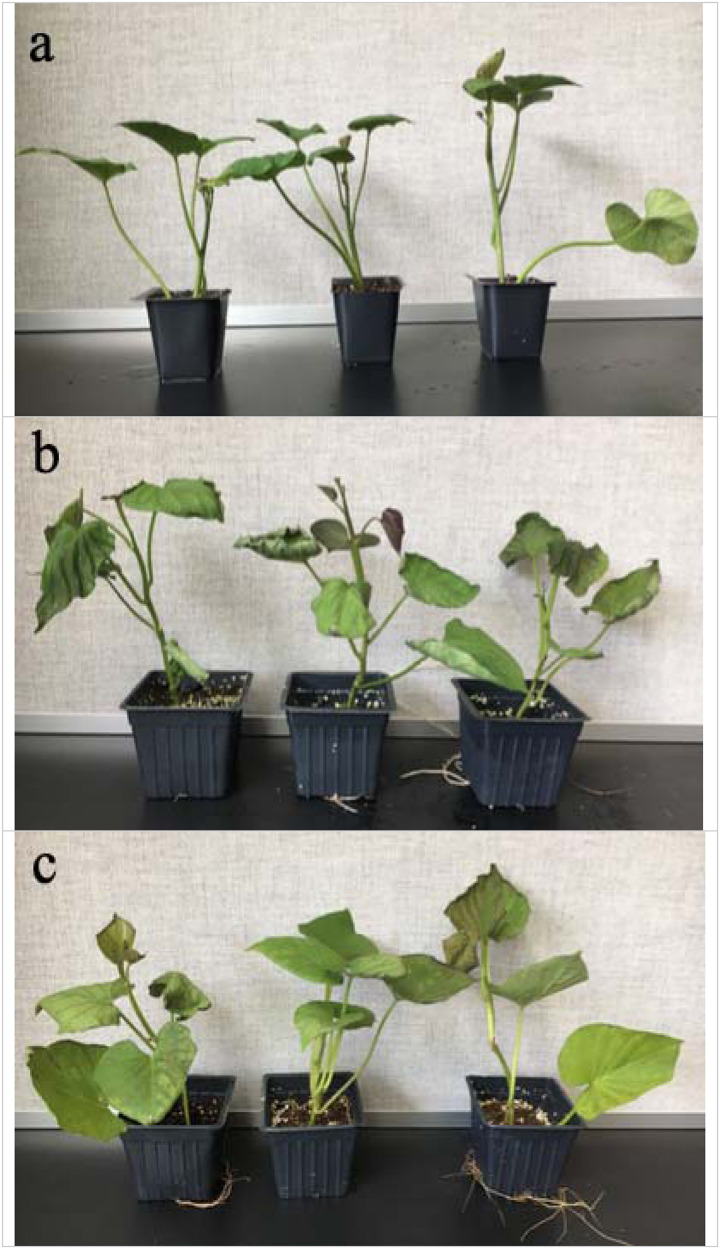
Effects of cold and heat stress on plant phenotype after 48 h of cold and heat treatment. **(a)** Control group. **(b)** 4°C cold stress group. **(c)** 47°C heat stress group. Compared to the high temperature stress, sweetpotato is more sensitive to the chilling stress.

Compared with the control group, the treated sweetpotato seedlings showed obvious wilting after 48 h of chilling treatment, and the leaves of the heat-treated seedlings turned brown and curl slightly (Figure 1). Sweetpotato plants were more sensitive to chilling stress than high temperature heat stress.

### Chilling and Heat Stress-Induced Oxidative Stress and the Related Biochemical Changes in Sweetpotato

**FIGURE 2:**
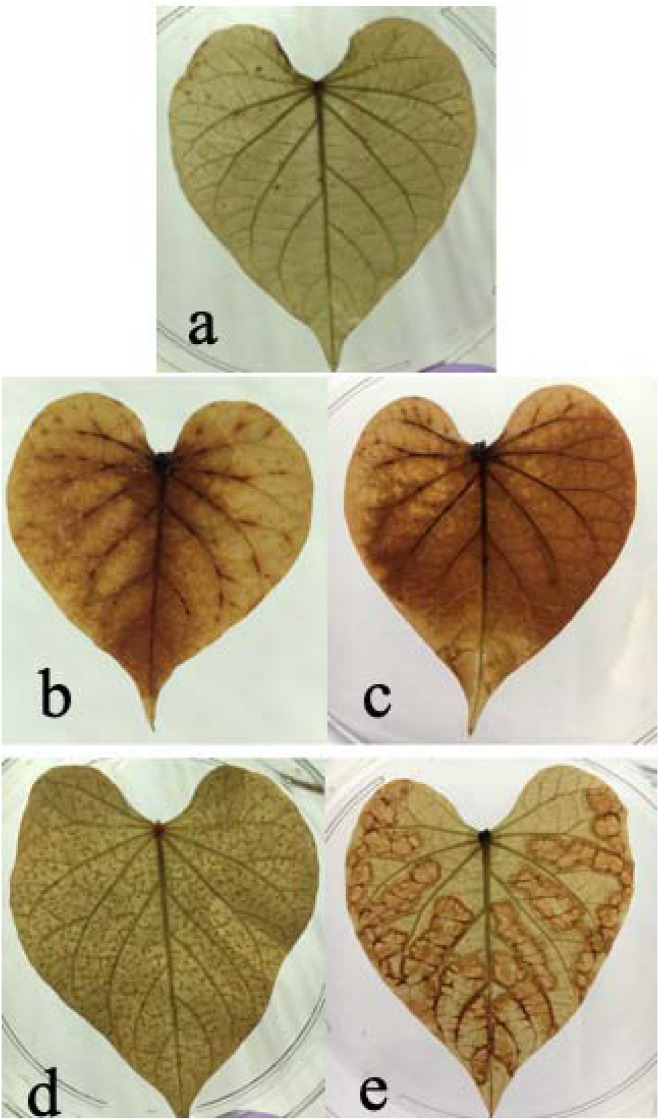
Effects of cold and heat stress on H_2_O_2_ synthesis in leaves after 48 h of cold and heat treatment. **(a)** Control group. **(b)** 4°C cold treatment for 24 h. **(c)** 4°C cold treatment for 48 h. **(d)** 47°C heat treatment for 24 h. **(e)** 47°C heat treatment for 48 h. The results are based on DAB staining as described in the “Materials and Methods” section.

In this study, we found both chilling and high temperature treatment resulted in H_2_O_2_ accumulation in sweetpotato leaves, evidenced by the leaves change color from green to brown and there are many brown spots on leaves. This phenomenon became worse as increasing treatment time. Compared with the control group, there were dense brown spots on the leaves after 24 h of heat stress. After 48 h of stress, there were obvious brown patches in the mesophyll area, indicating that the content of H_2_O_2_ increased under heat stress. Similarly, compared with the control group, there was a diffuse brown color on the leaves at 24 h after chilling stress, and more brown dispersion after 48 h, indicating that chilling caused an increase in H_2_O_2_ concentration in the leaves (Figure 2). It seems the H_2_O_2_ accumulation varied between chilling and heat treatment. For heat treatment, the H_2_O_2_ accumulation is worse in the edge of leaves than that in the middle of the leaves. For chilling stress, it seems that H_2_O_2_ accumulation started from the middle of leaves and then spread out the entire leaves.

**FIGURE 3:**
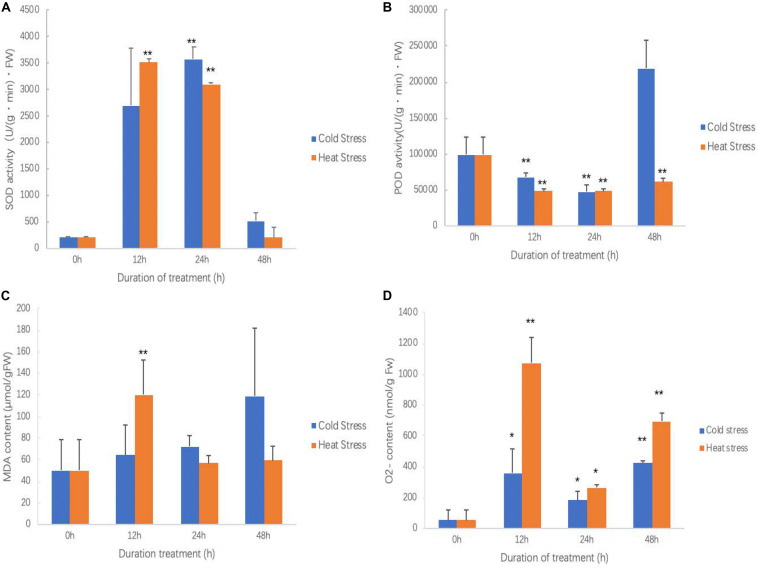
Effects of cold and heat stress on physiological indexes in sweetpotato leaves after cold and heat treatment. **(A)** SOD activities. **(B)** POD activities. **(C)** MDA contents. **(D)** O_2_^–^ contents. Five biological replicates were run for each treatment and control. Each treatment and control had five biological replicates for each measured trait at each time points. ANOVA was performed to analyze the significance between different treatment and controls at each time point. * presents significant difference between the treatment and the control with *p* < 0.05. ** presents extremely significant difference between the treatment and the control with *p* < 0.01.

As treatment going, the SOD activity of both chilling and heat treatment was increased first and then decreased (Figure 3A). At 24 h of chilling treatment, SOD activity reached the top and was extreme significantly higher than that in other times. For high temperature treatment, between 12 and 24 h, SOD activity reached the highest and was extreme significantly higher than that in 0 and 48 h of treatments. No matter for chilling treatment or high temperature treatment, after 48 h, SOD activity returned the levels of start points.

No matter in chilling stress or high temperature stress, POD activities were inhibited (Figure 3B). POD activity was continued to decrease after 12 h of chilling treatment; however, it seems that POD activity was recovered at 48 h. POD activity reached to the bottom at 12 h of high temperature treatment, and then slowly increased; however, it did not reach the level of controls.

During the chilling treatment, as increasing treatment time, leaves accumulated more MDA, but it did not reach a significant level (Figure 3C). However, high temperature treatment quickly resulted in MDA accumulation and reached to the highest at 12 h and then it was significantly reduced (Figure 3C). This suggests that high temperature quickly caused plant cell membrane lipid peroxidation; however, the chilling stress is a slow process. This result agrees with the results of ROS accumulation in which high temperature produced more ROS and leaves damaged more (Figure 2).

It has similar pattern for both chilling and high temperature stresses for inducing O_2_^–^ production (Figure 3D). Both chilling and high temperature treatment promoted sweetpotato cells to generate O_2_^–^ ROS at all observed time points. It also clearly saw that O_2_^–^ generation was very quickly and soon it reached the highest at 12 h of treatment; after that, at 24 h of treatment, O_2_^–^ content was decreased and then increased again at 48 h. This may be the physiological response of sweetpotato to chilling and high temperature stresses.

### Expression Profiles of Selected MiRNA and Their Target Genes Under Normal Condition in Sweetpotato

**FIGURE 4:**
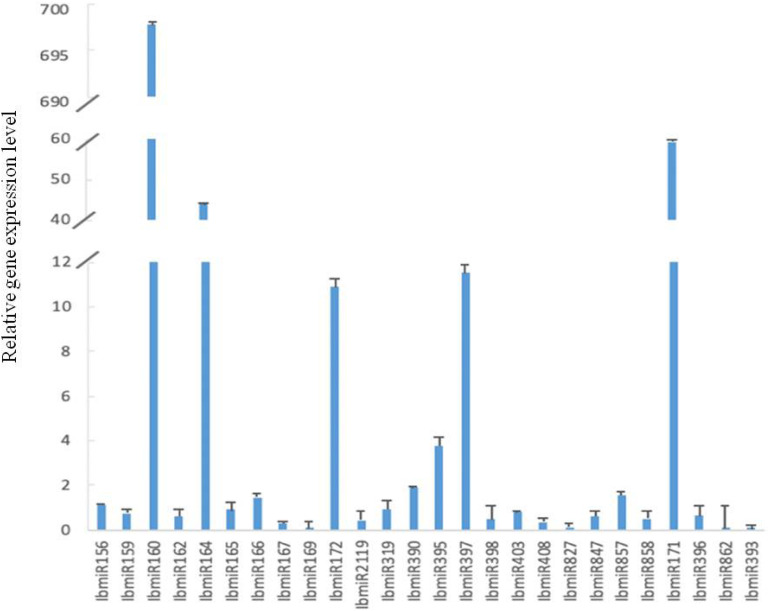
Expression profiles of 26 conserved miRNAs in leaves of sweetpotato under normal condition after 48 h of treatment. Relative gene expression level represented the relative expression of an individual miRNA gene compared with the reference gene *elf*. Five biological replicates and three technical replicates were run for each treatment and control.

All 26 tested miRNAs were expressed in sweetpotato leaves (Figure 4). However, their expression levels were different with a big range. Compared with the reference gene, the range from lower expression by 4.12 × 10^–6^ folds to higher expression by 2.56 × 10^5^ folds. Among these 26 miRNAs, the expression levels of 10 miRNAs (IbmiR156, IbmiR160, IbmiR164, IbmiR166, IbmiR172, IbmiR390, IbmiR395, IbmiR397, IbmiR857, IbmiR171) were higher than that of the reference gene; the rest 16 miRNAs (IbmiR159, IbmiR162, IbmiR165, IbmiR167, IbmiR169, IbmiR2119, IbmiR319, IbmiR398, IbmiR403, IbmiR408, IbmiR827, IbmiR847, IbmiR858, IbmiR396, IbmiR862, IbmiR393) were expressed lower than that of reference gene (Figure 4). The five miRNAs with highest expression levels were IbmiR160, IbmiR171, IbmiR164, IbmiR397, and IbmiR172. The 5 miRNAs with lowest expression levels were IbmiR393, IbmiR862, IbmiR169, IbmiR827, and IbmiR167.

**FIGURE 5:**
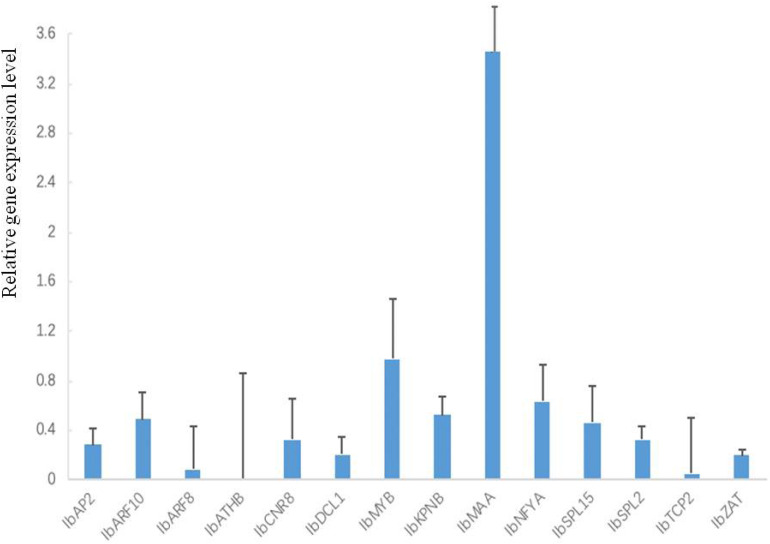
Expression profiles of 14 target genes in leaves of sweetpotato under normal condition after 48 h of treatment. Relative gene expression level represented the relative expression of an individual miRNA gene compared with the reference gene *elf*. Five biological replicates and three technical replicates were run for each treatment and control.

All 14 tested target genes were also expressed. Among them, *IbMAA* was highly expressed whereas the other 13 (*IbAP2*, *IbARF10*, *IbARF8*, *IbATHB*, *IbCNR8*, *IbDCL1*, *IbMYB*, *IbKPNB*, *IbNFYA*, *IbSPL15*, *IbSPL2*, *IbTCP2*, *IbZAT*) were expressed lower than the average (Figure 5).

### Chilling and Heat Stresses Altered the Expression of MiRNAs and Their Targets

**FIGURE 6:**
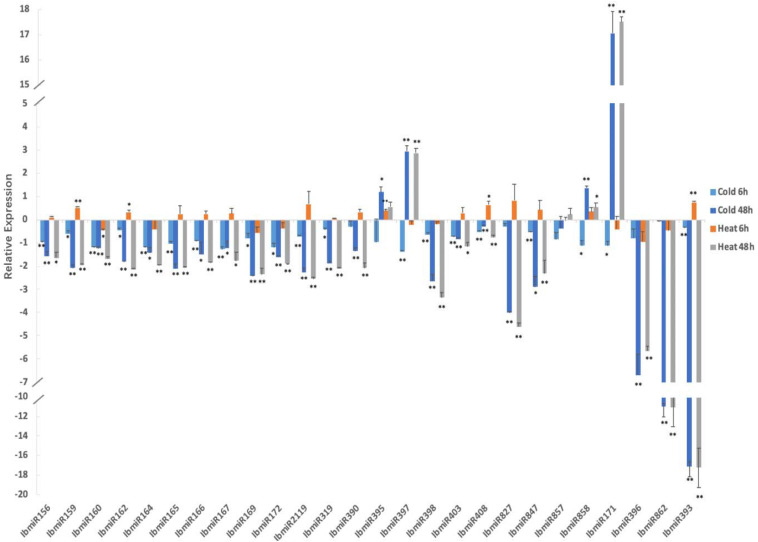
Chilling and heat stress induced the aberrant expression of miRNAs in sweetpotato leaves. Leaves were collected after 6 and 48 h of treatment. Five biological replicates and three technical replicates were run for each treatment and control. ANOVA was performed to analyze the significance between different treatment and controls at each time point. *Presents significant difference between the treatment and the control with *p* < 0.05. **Presents extremely significant difference between the treatment and the control with *p* < 0.01.

Chilling stress significantly altered the expression of 26 tested miRNAs (Figure 6). After 6 h of chilling treatment, the expression of seven miRNAs (IbmiR159, IbmiR162, IbmiR169, IbmiR172, IbmiR319, IbmiR858, IbmiR171) was significantly lower in the treatment groups than that in the control group; 13 miRNAs (IbmiR156, IbmiR160, IbmiR164, IbmiR165, IbmiR166, IbmiR167, IbmiR2119, IbmiR397, IbmiR398, IbmiR403, IbmiR408, IbmiR847, IbmiR393) were expressed extremely significantly lower than that in the control group. After 48 h of chilling treatment, the expression of miR395 was significantly higher than that in the un-treated sweetpotato leaves, the expression levels of 3 miRNAs (miR397, miR858, and miR171) were extreme significantly higher than that in the un-treated sweetpotato leaves; four miRNAs (miR164, miR166, miR167, and miR847) were expressed significantly lower than that in the un-treated sweetpotato leaves whereas 17 miRNAs (IbmiR156, IbmiR159, IbmiR160, IbmiR162, IbmiR165, IbmiR169, IbmiR172, IbmiR2119, IbmiR319, IbmiR390, IbmiR398, IbmiR403, IbmiR408, IbmiR827, IbmiR396, IbmiR862, IbmiR393) were expressed highly significantly lower than that in the un-treated sweetpotato leaves.

High temperature stress also significantly altered the expression of 26 tested miRNAs (Figure 6). After 6 h of high temperature treatment, two miRNAs (miR162 and miR408) were expressed significantly higher than that in the un-treated sweetpotato leaves, the expression levels of three miRNAs (miR159, miR395 and miR393) were extreme significantly higher than that in the un-treated sweetpotato leaves. miR160 was expressed significantly lower than that in the un-treated sweetpotato leaves. After 48 h of high temperature treatment, among the 26 tested miRNAs, miR858 was expressed significantly higher than that in the un-treated sweetpotato leaves, the expression of two miRNAs (miR397 and miR171) were extreme significantly higher than that in the un-treated sweetpotato leaves; three miRNAs (miR156, miR167, and miR403) were significantly lower than that in the un-treated sweetpotato leaves, and 18 miRNAs (IbmiR159, IbmiR160, IbmiR162, IbmiR164, IbmiR165, IbmiR166, IbmiR169, IbmiR172, IbmiR2119, IbmiR319, IbmiR390, IbmiR398, IbmiR408, IbmiR827, IbmiR847, IbmiR396, IbmiR862, IbmiR393) were expressed highly significantly lower than that in the un-treated sweetpotato leaves.

**FIGURE 7:**
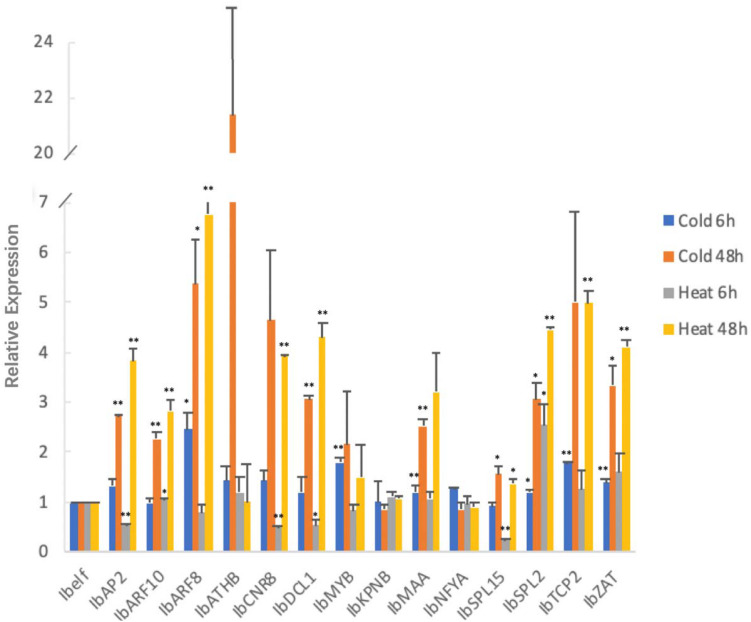
Chilling and heat stress induced the aberrant expression of miRNA target genes in sweetpotato leaves. Leaves were collected after 6 and 48 h of treatment. Five biological replicates and three technical replicates were run for each treatment and control. ANOVA was performed to analyze the significance between different treatment and controls at each time point. *Presents significant difference between the treatment and the control with *p* < 0.05. **Presents extremely significant difference between the treatment and the control with *p* < 0.01.

MicroRNA targets also responded to chilling and high temperature stress at a different way (Figure 7). After 6 h of chilling stress, among the 14 tested target genes, *IbARF8* and *IbSPL2* were expressed significantly higher than that in the untreated control group, and the expression levels of four genes (*IbMYB*, *IbMAA*, *IbTCP2*, and *IbZAT*) were extreme significantly higher than that in the un-treated sweetpotato leaves. After 48 h of chilling stress, four genes (*IbARF8*, *IbSPL15*, *IbSPL2*, and *IbZAT*) were expressed significantly higher than that in the un-treated sweetpotato leaves, and the expression levels of four genes (*IbAP2*, *IbARF10*, *IbDCL1*, and *IbMAA*) were extreme significantly higher than that in the un-treated sweetpotato leaves.

After 6 h of high temperature treatment, the expression levels of *IbARF10* and *IbSPL2* were significantly higher than that in the un-treated sweetpotato leaves whereas *IbDCL1* was expressed significantly lower than that in the un-treated sweetpotato leaves. *IbAP2*, *IbCNR8*, and *IbSPL15* were expressed highly significantly lower than that in the un-treated sweetpotato leaves. After 48 h of high temperature treatment, the expression level of *IbSPL15* was significantly lower than that in the untreated control groups; eight target genes (*IbAP2*, *IbARF10*, *IbARF8*, *IbCNR8*, *IbDCL1*, *IbSPL2*, *IbTCP2*, and *IbZAT*) were extremely significantly higher than that in the un-treated sweetpotato leaves.

### MiRNA-Target Shows Reverse Correlation Under Stress Conditions

**FIGURE 8:**
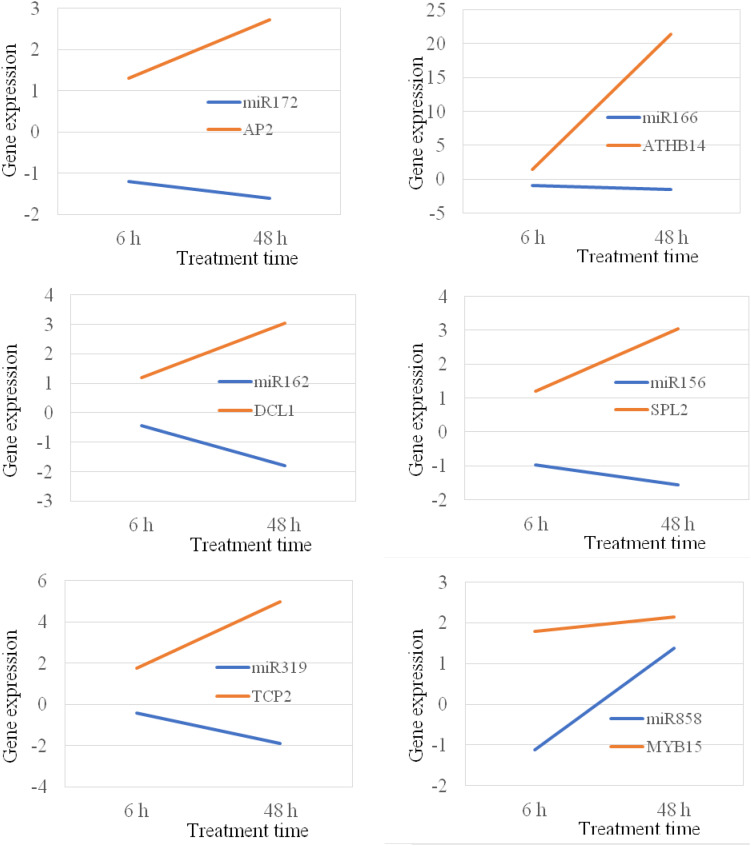
Position and negative correlations between the expression of miRNAs and their target genes under chilling stress.

**FIGURE 9:**
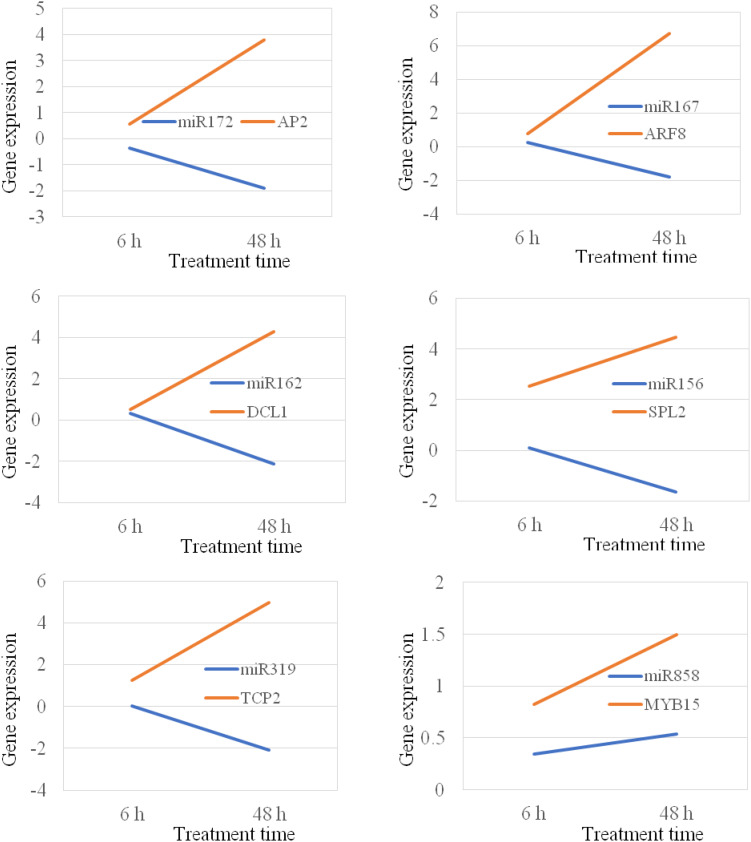
Position and negative correlations between the expression of miRNAs and their target genes under heat stress.

MicroRNAs negatively regulate gene expression by binding to their target mRNAs for mRNA clavage and/or translation inhibition. In plant, the majority of miRNAs inhibit gene expression by mRNA breakdown at the binding sites (Zhang B. et al., 2007). Thus, the expression of miRNA and their target genes should be in a reverse manner. It means that if the expression of a miRNA gene is increased, the expression of its target gene should be decreased. In this study, we analyzed 14 miRNA targets that are targeted by 10 different miRNAs. Our study show that the majority of miRNAs, their expression were negatively correlated with the expression of their corresponding target genes under both chilling and heat stress conditions (Figures 8, 9); however, there were also several miRNA-target pairs show positive relationship between the expression of miRNAs and their targets (Figures 8, 9). This suggests that the gene regulation is a complicated mechanism in the cells, particularly under the stress condition. Except the miRNA-mediated gene regulation, there are also other regulation mechanisms, such as DNA methylation and feedback regulation, all of them affect gene expression. For the miRNA-mediated gene regulation, there is also a potential that two or more miRNAs regulate the expression of a same protein-coding gene. Thus, it is a common phenomenon that the expression of a miRNA is not always negatively correlated with the expression of its target genes. This is also observed in other plant species (Lopez-Gomollon et al., 2012).

## Discussion

Chilling and heat (high temperature) stress is one of main factors limiting crop growth, development and then yield and quality; it has been gradually gained people’s attention (Nozzolillo et al., 1990; Bharti and Khurana, 1997). During cold acclimation, it has been observed that cell membrane structure was considerably changed (Yoshida and Uemura, 1990; Kubacka-Zębalska and Kacperska, 1999). Exposure to hypothermia increased thylakoid membrane damage due to ROS production (Leipner et al., 1999; Pastori et al., 2000). As a key enzyme in ROS scavenging system, SOD promotes the disproportionation of superoxide into oxygen and H_2_O_2_, thus it reduces the peroxidation of membrane lipids, maintains the stability of cell membrane, and then removes it through different pathways (Bowler et al., 1992; Koca et al., 2006; Zhang F.-Q. et al., 2007; Zhang et al., 2011). In fact, even under appropriate conditions, ROS is still produced in plant cells, but due to the antioxidant system of plants that can clear the ROS to some extent, so it will not harm the plant cells. However, under adverse conditions, ROSs are produced in a large quantity, and the activities of various enzymes in the antioxidant system are inhibited, so ROS can cause damage to plants. SOD and POD are two important enzymes in the plant ROS clearance system. In the early stages of exposure to chilling and heat stress, plants produce excessive amounts of ROS, and SOD activity is enhanced rapidly to respond to the stress whereas is not sufficient to eliminate excess ROS. After 24 h of stress, the degree of peroxidation of plant membrane lipids has increased, which is beyond the self-regulation of plants. Under chilling stress, POD activity remained declining and increased after 24 h. Studies have shown that POD has a relatively lagging reaction to low temperature due to the cross-effect of enzyme activity such as SOD (Zhang X. H. et al., 2015). Zou et al. (2007) and Zhu et al. (2007) also observed that POD activity increased under chilling stress. Our study is consistent with these results. This study show that both chilling and heat stress induced oxidative stress in sweetpotato seedlings. Under heat stress, the decrease of POD activity began to increase after 12 h, but it was still significantly lower than that in the untreated plants at 48 h of treatment, indicating that under heat stress, POD may not be the main protective enzyme in sweetpotato seedlings. H_2_O_2_ is a common ROS and it is widely existed in plant cells. However, when it accumulates in plant cells, ROSs cause oxidative damage and further affect other important traits. It is well demonstrated that abiotic stress induces the accumulation of ROSs, including H_2_O_2_ (Apel and Hirt, 2004). Our study also show the similar phenomenon, as extreme temperature treated, sweetpotato leaves generated H_2_O_2_.

MicroRNAs are an extensive class of small regulatory RNAs, which regulate gene expression at the post-transcriptional levels. miRNAs play an important and critical role in gene regulatory networks. miRNAs are widely existed and highly conserved in plant kingdom. Plant miRNAs can inhibit gene expression by directing mRNA cleavage and inhibiting translation of target transcripts (McConnell et al., 2001; Rhoades et al., 2002; Aukerman, 2003; Mallory et al., 2004; Baker et al., 2005). miRNAs are involved in a variety of biotic and abiotic stresses in plants, including pathogen infection, drought, chilling and heat stress (Zhang, 2015). Stress may lead to differential expression of certain miRNAs to regulate plant response to those stresses (He et al., 2014). To verify the differential expression of miRNAs and their target genes under chilling and heat stress, we can more deeply understand the regulation mechanism of sweetpotato after being stressed by ambient temperature. In this study, sweetpotato seedlings treated at different temperatures were selected as materials, and the expression levels of 26 miRNAs and 14 target genes were analyzed. Our study found that both chilling and heat stresses induced the altered expression of miRNAs in sweetpotato seedlings, suggesting that miRNAs play an role during sweetpotato seedling response to temperature stress. However, the exact regulatory mechanism of miRNAs still need more deep study in the future in sweetpotato. Our study also show that the majority of miRNAs and their target genes, such as IbmiR156 and its target *IbSPLs*, IbmiR159 and its target *IbMYB*, IbmiR160 and its target *IbARF10*, IbmiR167 and its target *IbARF8*, were negatively correlated with temperature stress, which was consistent with other crops. However, the expression of miRNAs and their target genes does not always follow a negative correlation. *DCL1* is a gene involved in miRNA maturation and function, and it is targeted by miR162. Therefore, there is also a negative regulatory mechanism between miR162 and *DCL1*. In this experiment, the expression level of IbmiR162 and its target gene *IbDCL1* is positively correlated under heat stress. This phenomenon is not surprised because gene regulation is a complicated gene network. Except a miRNA targets a protein-coding gene, other factors may also regulate *dcl1* gene expression.

MicroRNAs can regulate auxin receptor or response genes to regulate the auxin signaling pathway. Overexpressing the miRl67 target genes *ARF6* and *ARF8* regulated developmental abnormalities in *A. thaliana* and increased the occurrence of adventitious roots by regulating auxin signal pathway (Wu et al., 2006; Gutierrez et al., 2012). The *ARF* transcription factor regulates auxin-induced gene expression by binding to auxin in response to an activator. This study found that IbmiR160 and IbmiR167 acting on *ARF* transcription factors are up-regulated after chilling and heat stress, IbmiR160 and IbmiR167 may regulate sweetpotato response to chilling and heat stress through regulating auxin signal pathway.

Copper is an essential trace element involved in photosynthesis, oxidation and other physiological processes. After exposure to chilling and heat stress, some miRNAs that regulate the homeostasis of copper, such as IbmiR398 and IbmiR397, were differentially expressed. In *Arabidopsis*, miR398 is an important regulator of copper balance, which reduces copper flux to copper/zinc superoxide dismutase and flows to more essential biological processes to accommodate low copper stress (Abdel-Ghany and Pilon, 2008). In this study, the differential expression of IbmiR398 during chilling and heat stress suggests that IbmiR398 is involved in sweetpotato response to chilling and heat stress. Lignin is the main component of plant secondary cell wall, and its degradation is regulated by a blue copper oxidase-laccase. The two laccase-encoding carrot genes *DcLac1* and *DcLac2* showed the same expression pattern under chilling and heat stress, indicating that laccase can respond to chilling and heat stress by affecting the synthesis of lignin (Kazan, 2015). In this study, IbmiR397, that targets laccase gene, was differentially expressed during chilling and heat stress. The results showed that sweetpotato IbmiR398 and IbmiR397 can act on their target genes and caused imbalance of nutrients and metabolites in plant cells.

As a target gene of miR319, transcription factor *TCP4* activates the expression of the key gene *LOX2* in the jasmonic acid biosynthesis pathway, thereby regulating the signal pathway of the hormone jasmonic acid (Schommer et al., 2008). miRl56 target gene *SPL9* inhibits its expression by interacting with B-type ARR in *A. thaliana*, thereby blocking the cytokinin signaling pathway and causing its regenerative ability to decrease (Zhang T. et al., 2015).

**FIGURE 10:**
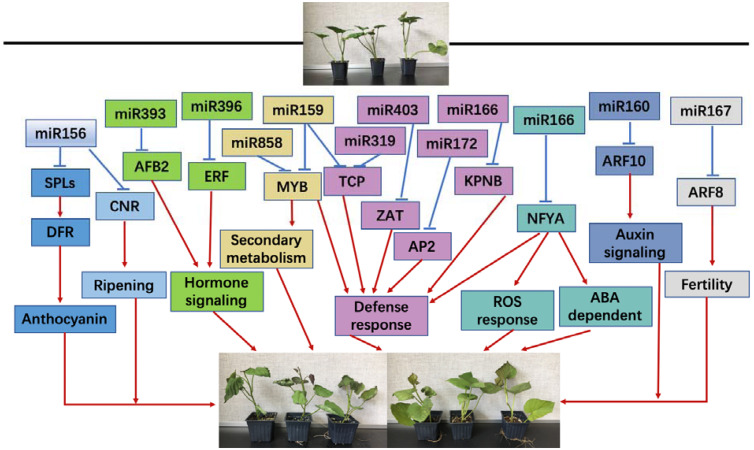
A proposed regulation network of miRNA response to chilling and heat stress in sweetpotato.

In conclusion, both chilling and heat stresses significantly induced physiological changes in sweetpotato seedlings, especially on the oxidative stress-related products and enzymes; during this biological process, miRNAs may play an important role evidenced by the altered changes on the expression of miRNAs and their targets. miRNA and its target genes may respond to chilling and heat stress by regulating sweetpotato growth and development by several biological processes, including anthocyanin, maturation process, secondary metabolism, defense response, ROS reaction pathway and ABA-dependent pathway (Figure 10).

## Data Availability Statement

The raw data supporting the conclusions of this article will be made available by the authors, without undue reservation, to any qualified researcher.

## Author Contributions

ZL and BZ conceived the experiments. JY, DS, DY, and TD conducted the experiments. JY, DY, YH, ZL, and BZ analyzed the results. ZL, HL, and BZ contributed to materials and analysis tools. JY, DS, ZL, and BZ wrote the manuscript. All authors contributed to the manuscript revision, read and approved the submitted version.

## Conflict of Interest

The authors declare that the research was conducted in the absence of any commercial or financial relationships that could be construed as a potential conflict of interest.
